# Design, synthesis, and biological evaluation of a multifunctional neuropeptide-Y conjugate for selective nuclear delivery of radiolanthanides

**DOI:** 10.1186/s13550-020-0612-8

**Published:** 2020-03-02

**Authors:** Adrien Chastel, Dennis J. Worm, Isabel D. Alves, Delphine Vimont, Melina Petrel, Samantha Fernandez, Philippe Garrigue, Philippe Fernandez, Elif Hindié, Annette G. Beck-Sickinger, Clément Morgat

**Affiliations:** 1 Department of Nuclear Medicine, University Hospital of Bordeaux, F-33076 Bordeaux, France; 2 University of Bordeaux, INCIA UMR 5287, F-33400 Talence, France; 3 CNRS, INCIA UMR 5287, F-33400 Talence, France; 4 Institute of Biochemistry, Faculty of Life Sciences, Leipzig University, Brüderstr. 34, 04103 Leipzig, Germany; 5 Institute of Chemistry & Biology of Membranes & Nano-objects (CBMN), CNRS UMR 5248, University of Bordeaux, F-33600 Pessac, France; 6 University of Bordeaux, Bordeaux Imaging Center, F-33000 Bordeaux, France; 7 Aix-Marseille University, INSERM, Institut National de la Recherche Agronomique, Centre de Recherche en Cardiovasculaire et Nutrition, 13385 Marseille, France; 8 Aix-Marseille University, Centre Européen de Recherche en Imagerie Médicale, 13005 Marseille, France

**Keywords:** ^161^Tb, Neuropeptide-Y, Sub-cellular delivery, Auger-emitter, Breast cancer

## Abstract

**Background:**

Targeting G protein-coupled receptors on the surface of cancer cells with peptide ligands is a promising concept for the selective tumor delivery of therapeutically active cargos, including radiometals for targeted radionuclide therapy (TRT). Recently, the radiolanthanide terbium-161 (^161^Tb) gained significant interest for TRT application, since it decays with medium-energy β-radiation but also emits a significant amount of conversion and Auger electrons with short tissue penetration range. The therapeutic efficiency of radiometals emitting Auger electrons, like ^161^Tb, can therefore be highly boosted by an additional subcellular delivery into the nucleus, in order to facilitate maximum dose deposition to the DNA. In this study, we describe the design of a multifunctional, radiolabeled neuropeptide-Y (NPY) conjugate, to address radiolanthanides to the nucleus of cells naturally overexpressing the human Y_1_ receptor (hY_1_R).

By using solid-phase peptide synthesis, the hY_1_R-preferring [F^7^,P^34^]-NPY was modified with a fatty acid, a cathepsin B-cleavable linker, followed by a nuclear localization sequence (NLS), and a DOTA chelator (compound pb12). In this proof-of-concept study, labeling was performed with either native terbium-159 (^nat^Tb), as surrogate for ^161^Tb, or with indium-111 (^111^In).

**Results:**

[^nat^Tb]Tb-pb12 showed a preserved high binding affinity to endogenous hY_1_R on MCF-7 cells and was able to induce receptor activation and internalization similar to the hY_1_R-preferring [F^7^,P^34^]-NPY. Specific internalization of the ^111^In-labeled conjugate into MCF-7 cells was observed, and importantly, time-dependent nuclear uptake of ^111^In was demonstrated. Study of metabolic stability showed that the peptide is insufficiently stable in human plasma. This was confirmed by injection of [^111^In]In-pb12 in nude mice bearing MCF-7 xenograft which showed specific uptake only at very early time point.

**Conclusion:**

The multifunctional NPY conjugate with a releasable DOTA-NLS unit represents a promising concept for enhanced TRT with Auger electron-emitting radiolanthanides. Our research is now focusing on improving the reported concept with respect to the poor plasmatic stability of this promising radiopeptide.

## Background

Targeted radionuclide therapy (TRT) uses radioisotopes linked to a vector (peptides, small molecules, antibodies, and other targeting agents) in order to target tumor cells or their natural environment and deliver cytotoxic radiations. Encouraging results have been obtained in metastatic neuro-endocrine tumors with [^177^Lu]Lu-DOTATATE, a somatostatin analog radiolabeled with lutetium-177 (^177^Lu), that targets SST_2_R, the somatostatin receptor subtype 2 [1]. Similar success is envisioned with ^177^Lu-labeled prostate specific membrane antigen (PSMA) inhibitors in patients with castration-resistant metastatic prostate cancer [2]. However, about 20% of these patients will not respond to therapy or rapidly relapse with regrowth of micrometastases [2]. It is therefore necessary to increase toxicity of TRT to tumor cells. Modeling studies have identified terbium-161 (^161^Tb) as a potential alternative to ^177^Lu to deliver higher cytotoxic radiation doses without kidney damage [3]. ^161^Tb is a radiolanthanide that is chemically similar to ^177^Lu but is expected to outperform ^177^Lu due to its emission spectra rich in Auger electrons [4, 5]. Recent preclinical findings confirm the higher tumor control of [^161^Tb]Tb-PSMA compared with [^177^Lu]Lu-PSMA [6, 7]. Due to the short range of Auger electrons (few micrometers), the efficacy of such Auger-electron emitters can be maximized if located inside or close to the nucleus. A nuclear delivery of ^161^Tb after cellular internalization could therefore significantly increase the dose deposited to the genomic DNA and enhance the cancer cell-killing effect of this radiolanthanide [8].

Our team works on improving the efficacy of TRT and extending applications to other targetable receptors that are overexpressed in tumors [9, 10]. One promising target is the human Y_1_ receptor (hY_1_R) which was found to be overexpressed in some tumors, such as estrogen receptor (ER)-positive breast cancer [11].

Here, we describe the combination of a full-length hY_1_R-targeting peptide with a releasable NLS-DOTA unit in order to deliver radiolanthanides for better efficacy.

## Materials and methods

### Synthesis of the novel pb12 NPY conjugate; its labeling with natural terbium (^159^Tb), or its radio-labeling with indium-111 (^111^In)

The synthesis of the new NPY-conjugates is summarized below. Additional details are available in supporting information.

**Fig. 1:**
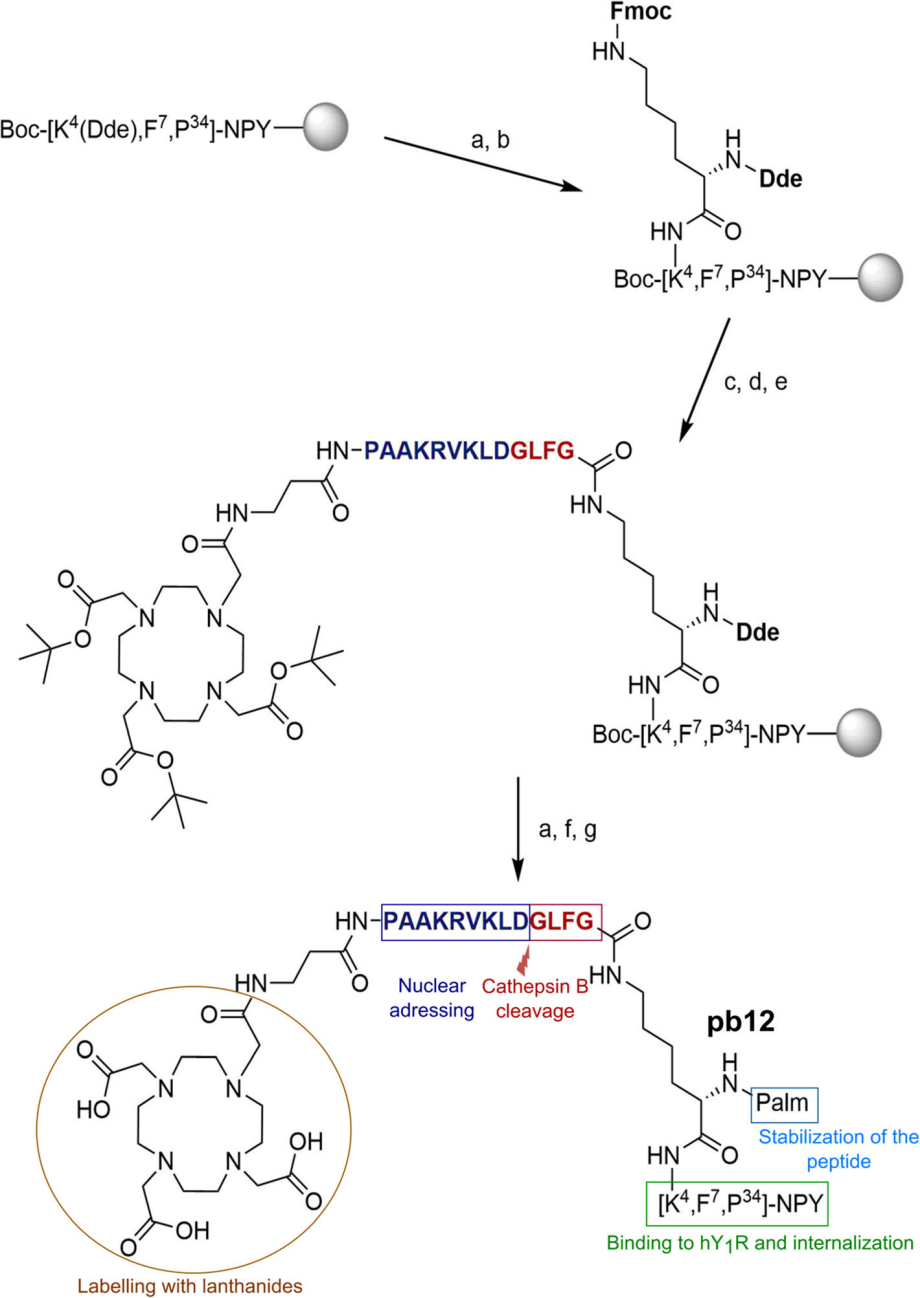
Synthesis scheme for the hY_1_R-targeted, multifunctional NPY conjugate pb12. Dde, 4,4-dimethyl-2,6-dioxocyclohex-1-ylidenethyl; SPPS, solid phase peptide synthesis; Fmoc, 9-fluorenylmethoxycarbonyl; DMF, dimethylformamide; HOBt, 1-hydroxybenzotriazole; DIC, *N,N*'-diisopropylcarbodiimide; NLS, nuclear localization sequence; DOTA, 1,4,7,10-tetraazacyclododecane-1,4,7,10-tetraacetic acid; TFA, trifluoroacetic acid; TA, thioanisole; TC, thiocresole; Palm, palmitic acid. The cathepsin B-cleavable linker is marked in red and the nuclear localization sequence in blue. a Dde cleavage, b coupling, c Fmoc cleavage, d extension with β-Ala NLS GLFG sequence, e DOTA coupling, f palm coupling, g full cleavage

For the DOTA-NLS-NPY conjugate pb12, newly developed in our laboratories, the backbone sequence of [F^7^,P^34^]-NPY was synthesized by robot-assisted SPPS using the Fmoc/*t*Bu strategy [12]. As modification point, the natural Lys^4^ in [F^7^,P^34^]-NPY was chosen. In previous studies, modification of Lys^4^ with toxic agents or carboranes did not change the activity and selectivity profile of the peptide [13, 14]. To facilitate side-specific modification in pb12, the *N*_*ε*_-amino group of Lys^4^ was orthogonally protected by Dde. After the removal of the Dde group with hydrazine, the amino acid Dde-Lys(Fmoc)-OH was coupled as branching unit to the *N*_*ε*_-amino group of Lys^4^ (Fig. 1). Subsequently, manual Fmoc deprotection was performed and the free *N*_*ε*_-amino group of the Lys branching unit could be extended with the amino acid sequence βAla-PAAKRVKLDGLFG by automated SPPS. GLFG thereby acts as cleavage site for the lysosomal protease cathepsin B [15], while PAAKRVKLD represents the NLS from the c-Myc protein [16]. β-Ala was introduced as short spacer. Next, the bifunctional chelating agent DOTA was manually coupled to the *N*-terminus of the β-alanine spacer with DIC/HOBt by using the protected building block DOTA-tris(*t*Bu)ester. Finally, the Dde protecting group at the *N*_*α*_-amino group of the Lys branching unit was removed, and palmitic acid was manually coupled with DIC/HOBt to yield conjugate pb12. For the control peptide pb13, the peptide backbone [F^7^,A^33^,P^34^,A^35^]-NPY was used and modification at Lys^4^ was performed as described for pb12. Replacement of the Arg^33^ and Arg^35^ residues in [F^7^,P^34^]-NPY with alanine was previously shown to delete the binding affinity of the peptide and is therefore suitable for the generation of a negative control peptide [17].

For first biological in vitro experiments, the DOTA-containing NPY conjugates pb12 and pb13 were labeled with ^nat^Tb or ^111^In as surrogate for radiolanthanides as described below:

The pb12 and pb13 conjugates (0.34 μmol) were dissolved in 500 μL of 0.4 M aqueous ammonium acetate solution (pH 5), and 100 μL of 0.01 M aqueous ^nat^TbCl_3_ solution were added. The mixture was incubated for 30 min at 37 °C and 500 rpm in a dry block heater and subsequently cooled to room temperature. Removal of salts was accomplished by using Amicon® Ultra-4 MWCO 3000 centrifugal filter units (Merck) or PD MidiTrap G-25 desalting columns (GE Healthcare, Chicago, IL, USA) to obtain conjugates [^nat^Tb]Tb-pb12 and [^nat^Tb]Tb-pb13.

For radiolabeling with ^111^In, ^111^InCl_3_ (Curium Netherlands B.V, ~ 250 MBq) was incubated at room temperature with 50 μg of DOTA-peptide (pb12 or pb13) in 500 μL of 0.1 M acetate buffer (pH 5) for 1.5 h under gentle agitation. The raw product was diluted to 3 mL with acetate buffer and purified on a PD-10 size exclusion column (GE Healthcare) according to the manufacturer’s instructions. Elution was performed with 3.5 mL of acetate buffer, and fractions of 0.5 mL were collected. The two fractions with the highest activities were pooled. Radiochemical purity was monitored by UV-radio RP-HPLC using a Phenomenex Luna C18 column (150 mm × 4.6 mm, 5 μm, 4 mL/min) with a linear gradient of 20–70% eluent B (ACN) in eluent A (0.1% (v/v) TFA in water) over 10 min and detection at *λ* = 220 nm.

### Determination of lipophilicity

The lipophilicity of ^111^In-labeled peptides was assessed by the water-octanol partition/distribution coefficient method as described in detail in supporting information.

### Assessment of cathepsin B-mediated cleavage of pb12

Two micromolar of pb12 were incubated with 0.74 U of recombinant cathepsin B for 0, 0.25, 0.5, 1, and 2 hours at 37 °C. Analysis was conducted by HPLC and peptide stability was monitored with UV-HPLC with and without the cathepsin B inhibitor CA-074.

Cell culture, hY_1_R, and cathepsin B expressions assessed using western blot and immunofluorescence on MCF-7 cells and cathepsin B activity of MCF-7 cells determined using ELISA assay are described in supporting information.

Receptor activation, receptor internalization, membrane integrity assay, PIXE experiment, and metabolic stability are also described in supporting information.

### Saturation radioligand binding assay

Saturation binding assays were conducted on hY_1_R-expressing MCF-7 breast cancer cells following the methodology of Ginj et al. used for [^111^In]In-DOTA-NLS-TOC [18]. The blocking agent used was the hY_1_R antagonist BIBP3226 (1 μM).

### Determination of specific internalization of radiolabeled NPY conjugates

The cellular accumulation of the ^111^In-labeled NPY conjugates was performed as described by Maschauer et al. [19], with only minor modifications (MCF-7 cells were seeded at a density of 1 million cells per well in 6-well plates and incubated overnight; 50 kBq of the respective ^111^In-labeled NPY conjugates was used, and membrane-bound radioligand was removed with 20 mM acetate buffer (pH 5)). Each peptide was tested in three independent experiments.

### Determination of cellular efflux of [^111^In]In-pb12

For efflux experiments, MCF-7 cells were seeded at a density of 1 million cells per well in 6-well plates and cultured overnight. A total of 50 kBq of [^111^In]In-pb12 (with or without pre-incubation of the efflux inhibitor probenecid at a concentration of 10 μM) was added to the medium and the cells were incubated (in triplicates) for 1 h at 37 °C. Three minutes before the end of the incubation time, internalization was stopped on ice, and the supernatant was removed. Each well was washed with 1 mL of ice-cold PBS. The membrane-bound fraction was retrieved in 2 mL sodium acetate buffer (20 mM, pH 5) for 2 min; each well was rinsed a second time with 1 mL ice-cold PBS, and fresh culture medium was added. At each time point (0.5, 1, 2, 4, and 24 h), the efflux was stopped by collecting the medium and washing cells twice with ice-cold PBS. Finally, cells were treated with NaOH (1 M). The radioactivity of the collected culture medium supernatant, the PBS wash fractions, and the total internalized fraction were measured in a gamma counter. The experiment was performed three times independently.

### Determination of nuclear uptake of ^111^In

To determine the amount of radioactivity in the nucleus, we proceeded similar to the radioligand internalization experiments. As additional control, the cathepsin-B inhibitor CA-074 Me was pre-incubated at a concentration of 10 μM for 1 h in selected wells. Instead of adding NaOH at the last step, 500 μL of trypsin was added to each well and incubated for 3 min at 37 °C. The content of each well was then centrifuged (5 min, 1800×*g*, 4 °C). The supernatant was removed; 1 mL of hypotonic buffer was added and incubated for 15 min on ice. Subsequently, the samples were again centrifuged (5 min, 1800×*g*, 4 °C), and the resulting supernatant corresponds to the cellular fraction, and the pellet corresponds to the nuclear fraction. Both fractions were separated and measured in a gamma counter. Finally, the percentage of radioactivity in the nucleus relative to the overall specifically internalized radioactivity or the total amount of used radioactivity was calculated. Experiments were independently performed at least two times in triplicates.

### In vivo [^111^In]In-pb12 SPECT/CT imaging

The MCF-7 tumor model was established as described by Hofmann et al [17]. Whole body SPECT/CT scans (duration 17 s each) of mice (23.5 ± 4.2 g, *n* = 3) bearing MCF-7 xenografts injected with [^111^In]In-pb12 (2.5 ± 0.2 MBq, 0.77 ± 0.07 μg, 71.7 ± 2.2 μL, 0.1 ± 0.01 nmol) in the tail vein were performed using a NanoSPECT/CT Plus (Mediso Medical Imaging System Ltd) at 12, 20, 29, 37, 46, 54, 63, 71, 80, 88, 96, 104, 240, and 1440 minutes post-injection. A second group of three mice were pre-injected with 50 μg (7.8 nmol) of pb12; 25 minutes before injection of [^111^In]In-pb12 (one mouse died at 2 h). CT parameters were X-ray, 45 kVp, exposition 500 ms, binning 1:4, and projection 180. Aperture was pinhole 102 Rat STD, whole body 2.2 mm. Region of interest (ROI) was manually drawn on reconstructed images using the Nucline software (v1.02) on several organs (MCF-7 tumors, lungs, heart, kidneys, liver, stomach, brain, bones, and muscle) at each time point, and data were expressed as percentage of injected activity per volume of tissue (%IA/mm^3^). SPECT and CT dicom files were fused using PMOD v3.5 (PMOD Technologies Ltd., Switzerland)

## Results

### Chemistry

Peptide conjugates were prepared by a combination of automated and manual SPPS (Fig. 1). The sequences and the names of the generated compounds are listed in Table 1. All compounds were highly pure (> 95%) (see supplemental Table 1 and supplemental Figure 1).

**Table 1:**
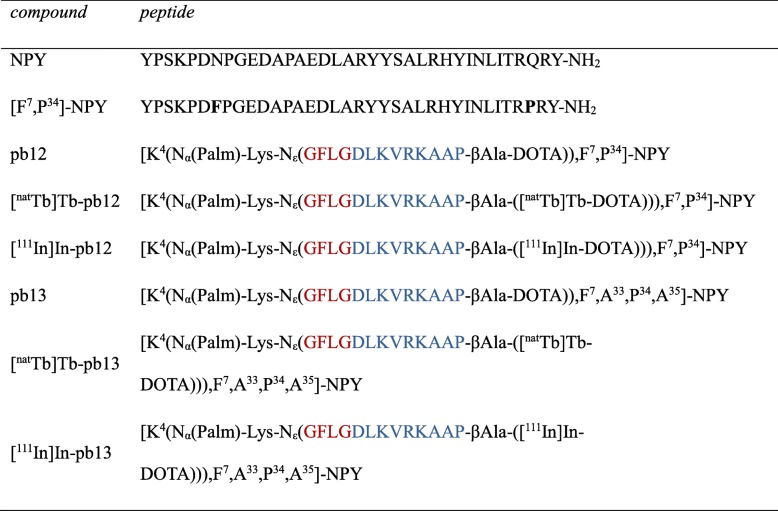
Nomenclature of the prepared NPY conjugates pb12, [^nat^Tb]Tb-pb12, [^111^In]In-pb12, and the non-binding control peptides pb13, [^nat^Tb]Tb-pb13, and [^111^In]In-pb13

The cathepsin B-cleavable linker is marked in red and the c-Myc nuclear localization sequence in blue. *Palm* palmitic acid, *DOTA* 1,4,7,10-tetraazacyclododecane-1,4,7,10-tetraacetic acid

### Characterization of pb12 and [^nat^Tb]Tb-pb12 compared with the control compounds pb13 and [^nat^Tb]Tb-pb13

#### Cleavage of pb12 by cathepsin-B

**Fig. 2:**
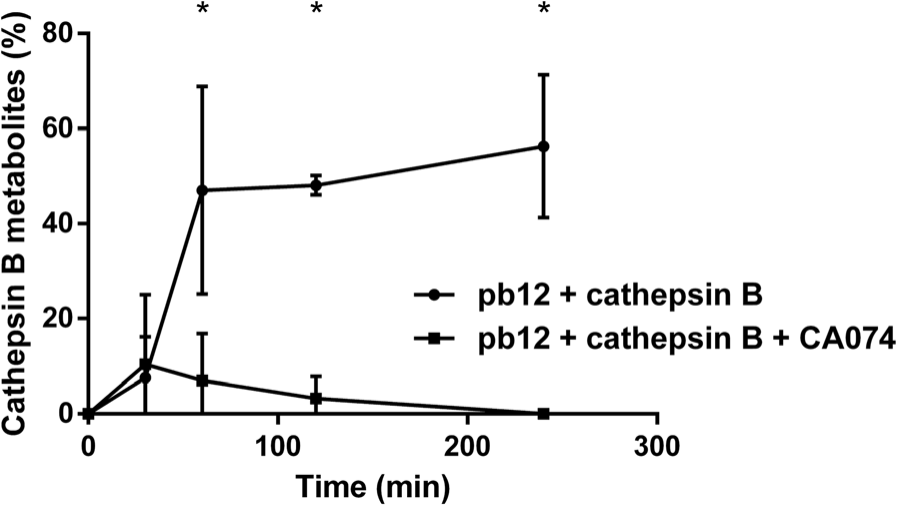
Kinetics of cathepsin B metabolite formation with and without the cathepsin B inhibitor CA074

The pb12 peptide cleavage mediated by recombinant cathepsin B was investigated in vitro and monitored using UV-HPLC as first proof of concept. Cathepsin B metabolite formation increases over time and was significantly inhibited by the cathepsin B inhibitor CA074 (Fig. 2)

#### Affinity, receptor activation, and internalization

**Fig. 3:**
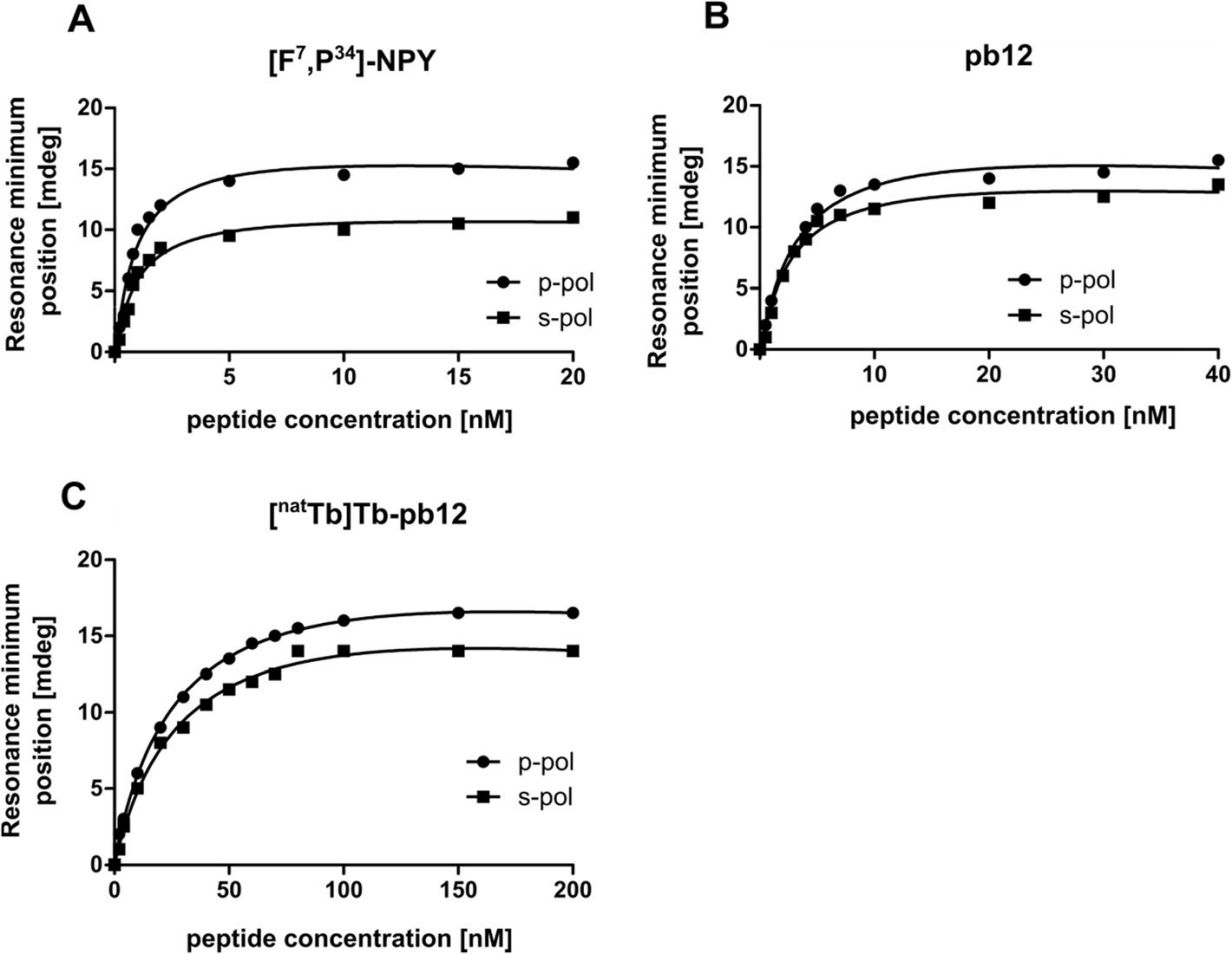
Affinity of [F^7^,P^34^]-NPY (control, **a**), pb12 (**b**), and [^nat^Tb]Tb-pb12 (**c**) determined using PWR on MCF-7 cell fragments. p-pol stands for the light component that is perpendicular to the sensor surface and so the lipid membrane, and s-pol stands for the light axis component that is parallel

The specific receptor binding of the non-radioactive conjugates pb12 and [^nat^Tb]Tb-pb12 (Fig. 3) and the control peptides pb13 and [^nat^Tb]Tb-pb13 (Supplemental Figure 5) were investigated on MCF-7 cells along with the reference compound [F^7^,P^34^]-NPY. First, saturation binding curves were measured by plasmon-waveguide resonance (PWR) spectroscopy. The pb12 conjugate displayed only a slightly decreased affinity compared with [F^7^,P^34^]-NPY (Fig. 3a) with a K_D_ of 3.1 ± 0.8 nM (Fig. 3b and Table 2). Labeling with ^nat^Tb, however, led to a ten-fold decrease in binding affinity (K_D_ 30 ± 3.7 nM for [^nat^Tb]Tb-pb12, Fig. 3c) compared with the unlabeled pb12 molecule.

**Table 2:** Affinity values of NPY-conjugates to the hY_1_R

Compounds	K_D_ at the hY_1_R (nM)
[F^7^,P^34^]-NPY	0.94 ± 0.08
pb12	3.1 ± 0.8
[^111^In]In-pb12	5.1 ± 3.5
[^nat^Tb]Tb-pb12	30 ± 3.7
pb13	No specific binding
[^111^In]In-pb13	No specific binding
[^nat^Tb]Tb-pb13	No specific binding

For the non-affine control peptides pb13 and [^nat^Tb]Tb-pb13, no saturation binding up to a concentration of 1 μM could be observed (Supplemental Figure 5A and 5B).

As additional experiment, binding of pb12 and [^nat^Tb]Tb-pb12 to non-hY_1_R-expressing HEK293 cells was investigated and some non-specific membrane interaction was measured (Supplemental Figure 5E and 5F).

**Fig. 4.**
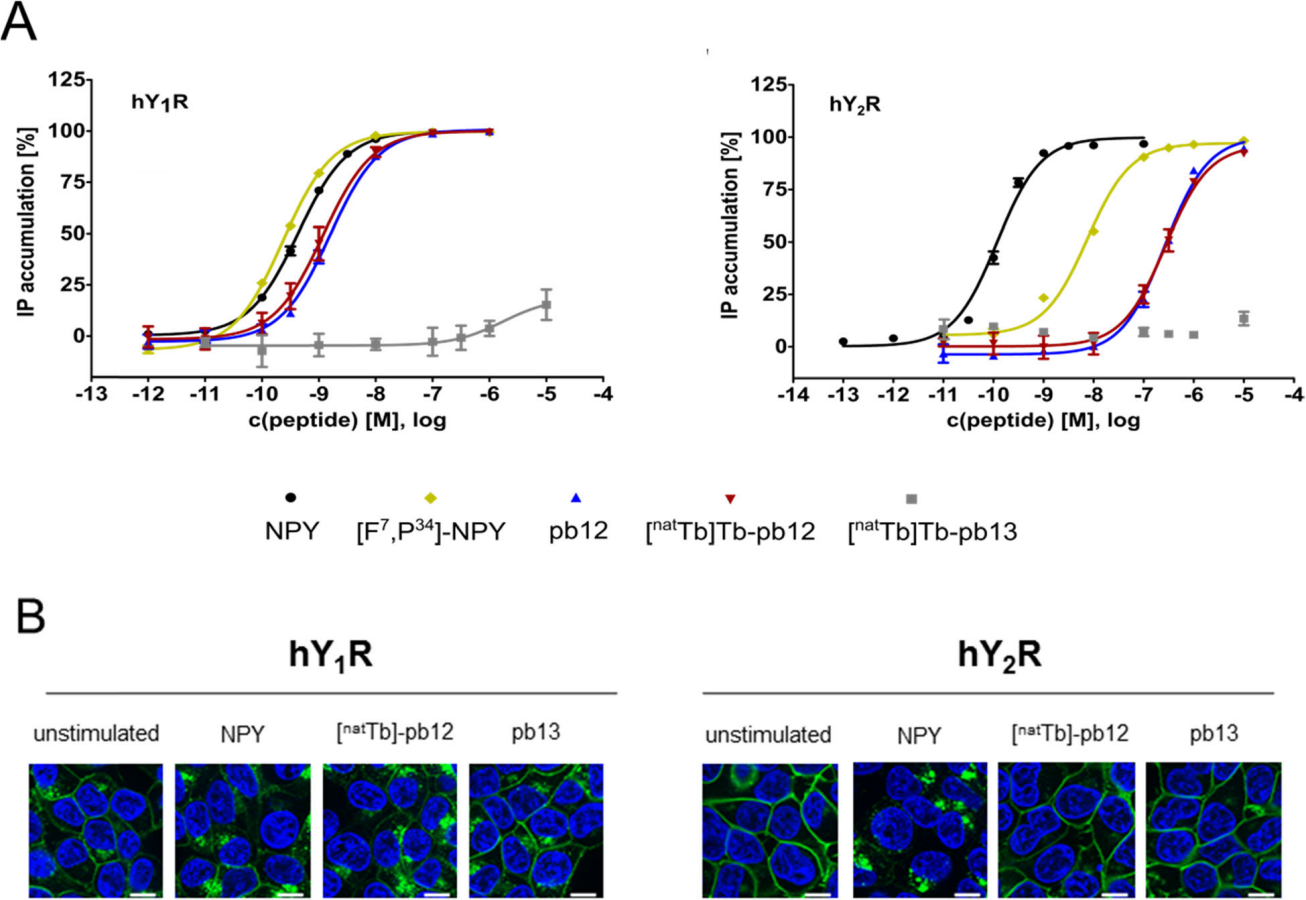
**a** NPY receptor activation profile by measurement of IP accumulation upon stimulation of the hY_1_R and hY_2_R with NPY conjugates NPY, [F^7^,P^34^]-NPY, pb12, [^nat^Tb]Tb-pb12, and [^nat^Tb]Tb-pb13. **b** Receptor internalization studies on HEK293 cells stably expressing the hY_1_R or hY_2_R fused to eYFP (green) following stimulation with [^nat^Tb]Tb-pb12 and pb13. Scale bar = 10 μm

Receptor activation of the hY_1_R and hY_2_R was investigated for the unlabeled DOTA-NLS-[F^7^,P^34^]-NPY conjugates pb12 and [^nat^Tb]Tb-pb12 on COS-7 cells stably co-expressing the hY_1_R or hY_2_R and the chimeric G protein Gα_Δ6qi4myr_ which switches the endogenous Gα_i_-coupled signaling pathway of the activated NPY receptors to the Gα_q_-coupled pathway, which results in the intracellular generation of inositol phosphates (Fig. 4a). Unlabeled conjugate pb12 is a full agonist at the hY_1_R and displayed only a slightly lower potency compared with NPY and [F^7^,P^34^]-NPY with an EC_50_ of 1.6 nM. Labeling with ^nat^Tb in pb12 did not change the nanomolar activity of the conjugate at the hY_1_R (EC_50_ 1.2 nM). Considering hY_2_R activation, pb12 and [^nat^Tb]Tb-pb12 were able to activate the hY_2_R only at very high concentration (10 μM). As regards the non-binding control [^nat^Tb]Tb-pb13, it was nearly inactive for the hY_1_R and completely inactive at the hY_2_R.

[^nat^Tb]Tb-pb12 and the unlabeled control peptide pb13 were also investigated in live-cell fluorescence microscopy studies for their capability to induce internalization of the hY_1_R and hY_2_R (Fig. 4b). In the unstimulated state, the fluorescence-tagged hY_1_R and hY_2_R (green) were predominantly localized in the plasma membrane. [^nat^Tb]Tb-pb12 was able to stimulate internalization of the hY_1_R comparable to the endogenous ligand NPY, whereas no internalization of the hY_2_R was observed (Fig. 4b), thus confirming the retained hY_1_R selectivity of [^nat^Tb]Tb-pb12 as determined in the receptor activation assay. The non-binding control pb13 was not able to induce internalization of either hY_1_R or hY_2_R.

#### Membrane integrity after application of pb12, [^nat^Tb]Tb-pb12 compared with the control peptide pb13 and non-exposed cells

Given the identification of some non-specific association of pb12 and [^nat^Tb]Tb-pb12 in experiments with non-hY_1_R-expressing HEK293 and potential impact in membrane organization and integrity, we also tested MCF-7 membrane integrity using a LDH release assay after application of the respective peptides (pb13 peptide and non-exposed cells were used as controls). Overall, LDH release was low for all peptides tested (around 5%) and comparable to control measurements (*p* > 0.05).

#### PIXE experiment using [^nat^Tb]Tb-p12

Because [^nat^Tb]Tb-pb12 displayed somewhat lower affinity than [^111^In]In-pb12 at the hY_1_R and to illustrate that the multivalent construct pb12 may enable delivery of terbium into MCF-7 cells, we performed PIXE experiments on MCF-7 cells pre-incubated with [^nat^Tb]Tb-pb12 as surrogate of [^161^Tb]Tb-pb12. After irradiation with 3 MeV protons and signal quantification, we were able to demonstrate that the pb12 construct is a suitable shuttle for terbium in hY_1_R-expressing cells. The amount of native terbium carried by [^nat^Tb]Tb-pb12 was significantly higher than in non-exposed cells (*p* = 0.041).

### Characterization of the radiolabeled [^111^In]In-pb12 and the control compound [^111^In]In-pb13

#### ^111^In radiolabeling

NPY conjugates pb12 and pb13 were subsequently labeled with the Auger electron- and γ-emitting ^111^In as surrogate for radiolanthanides because terbium isotopes are not yet widely available [20]. [^111^In]In-pb12 and [^111^In]In-pb13 were obtained in high radiochemical purity (> 92%), high enough for pre-clinical characterization. All preclinical characterizations were performed at a volumic activity of 20 MBq/mL except for in vivo imaging which was performed at 47 MBq/mL.

#### Hydrophilicity

By using the partition method, a LogD (pH 5) value of − 0.27 ± 0.19, a LogP (pH 7) value of − 1.20 ± 0.27, and a LogP (pH 7.4) value of − 0.99 ± 0.16 were found for [^111^In]In-pb12.

#### Affinity, internalization, efflux rate, and nuclear delivery of [^111^In]In-pb12 compared with [^111^In]In-pb13

[^111^In]In-pb12 and [^111^In]In-pb13 were investigated in a saturation radioligand binding assay on MCF-7 cells. [^111^In]In-pb12 displayed a retained, high-hY_1_R binding affinity (K_D_ 5.1 ± 3.5 nM, Supplemental Figure 5C and Table 2), comparable to unlabeled pb12. The control peptide [^111^In]In-pb13 showed no specific binding to the hY_1_R on MCF-7 cells (Supplemental Figure 5D) For easier comparison, Table 2 summarizes affinity values of all compounds evaluated in this work.

Next, the hY_1_R-mediated internalization of the radiolabeled conjugates [^111^In]In-pb12 and [^111^In]In-pb13 into MCF-7 cells was analyzed (Fig. 4a). For conjugate [^111^In]In-pb12, specific, time-dependent internalization into MCF-7 cells was observed with a maximum of 32.0 ± 9.7% of the total amount of used radiopeptide being internalized after 4 h (vs 11.7 ± 2.9% for the control peptide [^111^In]In-pb13 at 4 h, differences were significant at 1 h, 2 h and 4 h).

**Fig. 5:**
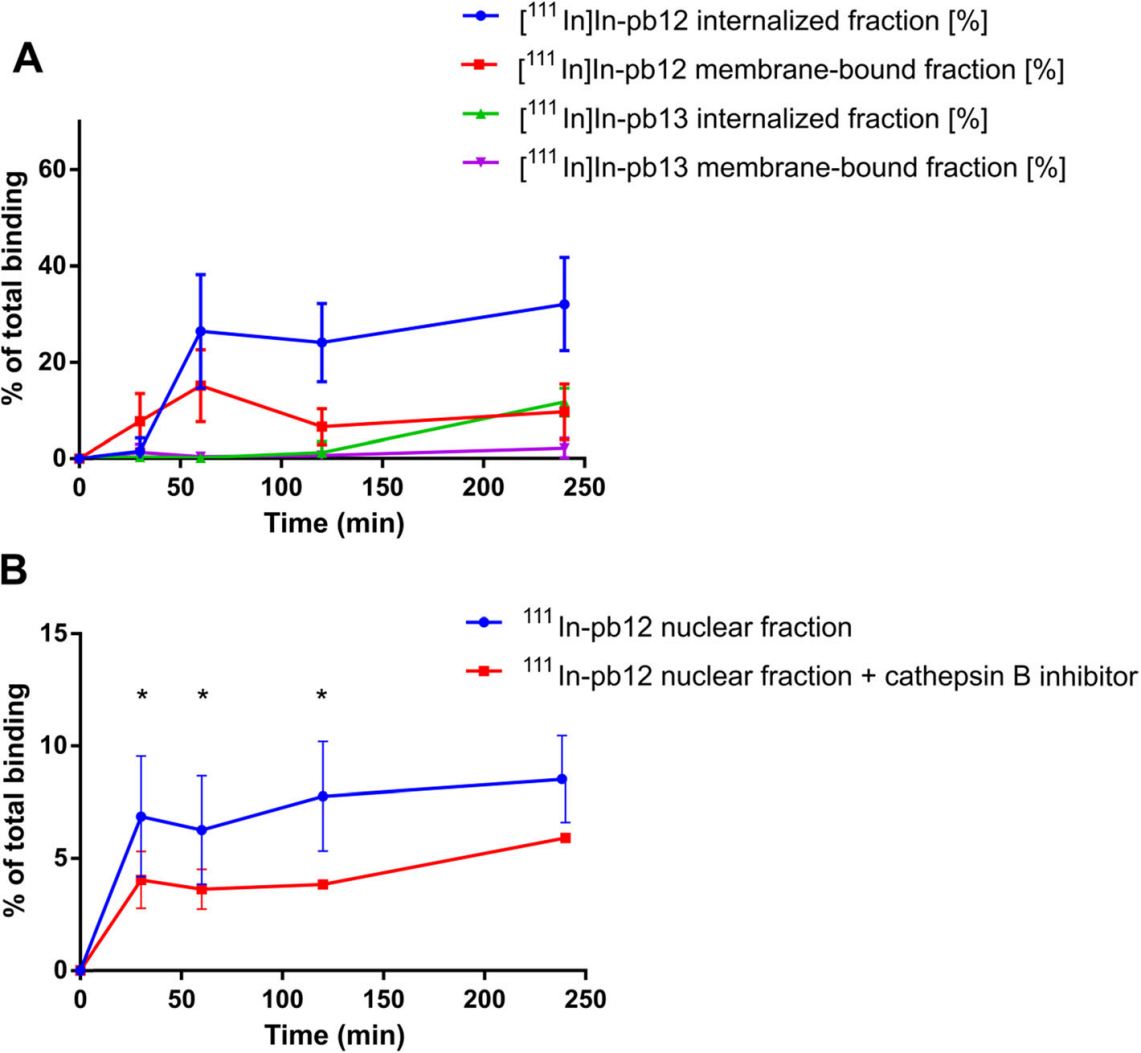
Determination of the internalized fraction and membrane-bound fraction of [^111^In]In-pb12 and control peptide [^111^In]In-pb13 (**a**) and nuclear fractions of [^111^In]In-pb12 with and without the cathepsin B inhibitor CA074Me (**b**) into endogenously hY_1_R-expressing MCF-7 cells. Data points represent the mean ± SD of at least two independent experiments

In another set of experiments, the nuclear delivery of ^111^In after internalization of [^111^In]In-pb12 into MCF-7 cells was investigated (Fig. 5b). Incubation of MCF-7 cells with the multifunctional conjugate [^111^In]In-pb12 resulted in a time-dependent nuclear uptake of ^111^In. After incubation for 2 h, a maximum of 7.7 ± 2.4% of the total radioactivity was found to be located in the nucleus (Fig. 5b). This value would yield to 24.1% of the internalized fraction addressed to the nucleus. A significant reduction of nuclear uptake of ^111^In at 3.8 ± 0.1% (*p* < 0.01) was observed when cells were pre-incubated with a cathepsin B inhibitor (Fig. 5b).

**Fig. 6:**
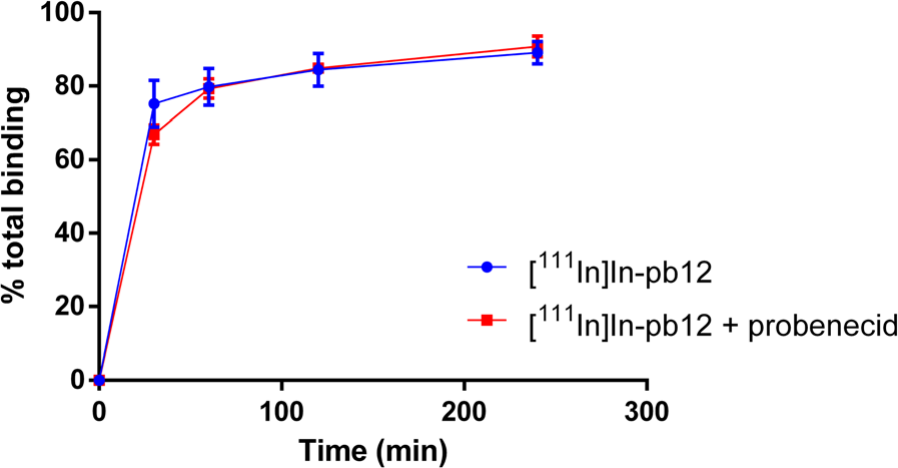
Investigation of the cellular efflux of [^111^In]In-pb12 with and without the efflux inhibitor probenecid. Data points represent the mean ± SD of three independent experiments

[^111^In]In-pb12 was further evaluated regarding cellular efflux. A high and fast efflux of radioactivity was found for [^111^In]In-pb12 (Fig. 6). Already after 30 min post internalization, 77 ± 5% of the total internalized radioactivity was detected in the medium outside the cells. This further increased to 88 ± 3% extracellular radioactivity after 4 h post internalization. However, the observed efflux was not inhibited by the efflux inhibitor probenecid (Fig. 6) and the control conjugate [^111^In]In-pb13 displayed a similar efflux profile (not shown).

#### Plasma stability of [^111^In]In-pb12

Metabolic stability of [^111^In]In-pb12 was evaluated in vitro in human plasma. Unfortunately, fast degradation of conjugate [^111^In]In-pb12 was observed, with ~20% of the initial radiopeptide left intact 1 h after incubation at 37 °C.

#### SPECT/CT imaging of [^111^In]In-pb12

**Fig. 7:**
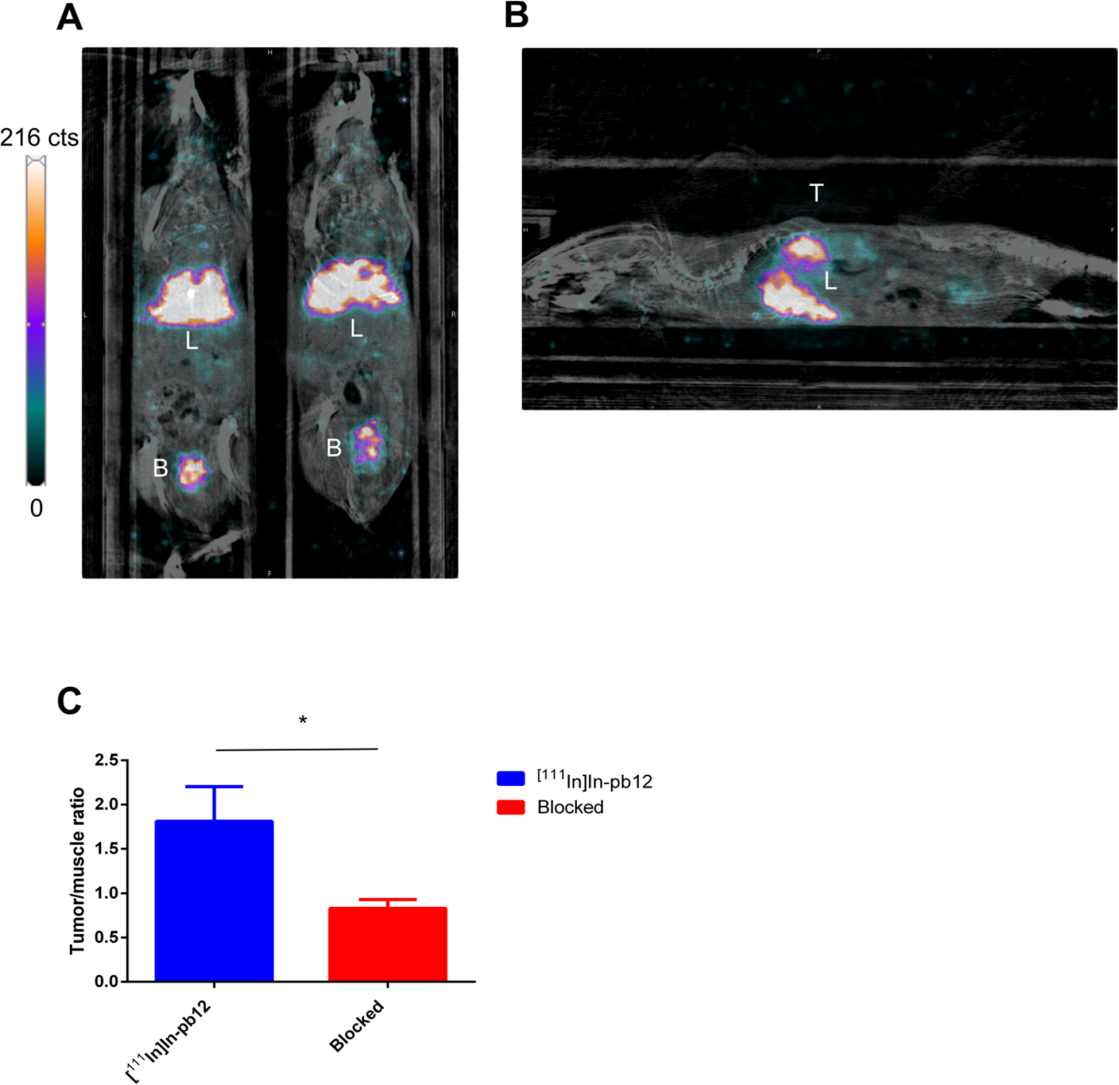
In vivo behavior of [^111^In]In-pb12 in MCF-7 xenograft mice. **a** Representative SPECT/CT coronal image at 12 min post-injection showing major elimination of [^111^In]In-pb12 by the liver and minor elimination by the bladder. **b** Representative SPECT/CT sagittal image at 12 min post-injection showing major elimination of [^111^In]In-pb12 by the liver. **c** Tumor/muscle ratio at 12 min post-injection of [^111^In]In-pb12 in MCF-7 xenografted mice determined on the SPECT/CT images. Co-injection of non-labeled pb12 (blocked group) significantly decreases the tumor/muscle ratio. L stands for liver, B means bladder and T stands for MCF-7 tumor

Given the low plasmatic stability of [^111^In]In-pb12, we focused on the first imaging time point at 12 minutes post-injection. [^111^In]In-pb12 showed preferential elimination via the liver (Fig. 7a, b; 0.205 ± 0.07%IA/mm^3^). Non-specific signal was also quantified in the heart (0.121 ± 0.03%IA/mm^3^) and the lungs (0.109 ± 0.05 %IA/mm^3^). Interestingly, low but specific uptake was noted in MCF-7 tumor (vs the blocked group, *p* = 0.001). Also, the tumor/muscle ratio was highly significantly reduced in the cold pb12-blocked mice (Fig. 7c, *p* = 0.01).

## Discussion

Targeted radionuclide therapy holds promise to deliver radiation to tumor cells over-expressing peptide receptors. In this work, we aimed to explore the feasibility of enhanced-TRT by using a subcellular active (radio) pharmaceutical able to deliver Auger-electron emitter like ^161^Tb, in tumors over-expressing the hY_1_R. Several strategies have been used to develop NPY ligands for imaging and therapy (non-peptide ligands, antagonistic-truncated peptides, or agonistic full-length peptides) with variable success [19, 21, 22]. The full-length hY_1_R-preferring peptide analog [F^7^,P^34^]-NPY allows selective delivery of cargos such as radioisotopes [12, 17, 23]. We described here the multivalent construct pb12 based on the hY_1_R-preferring [F^7^,P^34^]-NPY sequence, which rapidly internalizes into acidic endosomes after agonistic stimulation of the hY_1_R, modified with a cathepsin B-cleavable linker able to release a nuclear localization sequence (NLS) linked to a DOTA chelator to convey lanthanides into the nucleus using importins [24]. For therapy perspective, a profound in vitro characterization is needed to fully understand the biological mechanisms which might impact the efficacy. Receptor binding studies demonstrated that the advanced modification of [F^7^,P^34^]-NPY with a DOTA-NLS-GLFG unit and a fatty acid in pb12 did not influence the high hY_1_R affinity of the peptide. In addition, labeling with ^nat^Tb results in a decrease in affinity from 3 to 30 nM but the activity and biological behavior of the ^nat^Tb-labeled conjugate was hopefully maintained as demonstrated by PIXE experiments. pb12 was also radiolabeled with ^111^In as “surrogate” of lanthanides, and we showed that, in this case, the high hY_1_R affinity was preserved. The difference in affinity between the ^111^In-labeled version of pb12 and its ^nat^Tb counterpart illustrates the necessity of carefully determining affinity of metallated compounds for imaging and therapy. Importantly, these nanomolar affinity values compete well with other full-length peptides [17] or non-peptide ligands [21] and are better than those reported for truncated peptides [22].

We then looked for the hydrophilicity of [^111^In]In-pb12, and results showed that this compound is fairly hydrophilic. Addition of the lipophilic palmitic acid moiety might be responsible for the increased lipophilicity of our compound and lead to some non-specific binding of the peptide to the cell membrane. However, this interaction does not compromise cell membrane integrity as demonstrated by the LDH assay. Previous studies have shown that palmitoylation is an effective approach to enhance association of peptides with cell membrane and therefore increasing their proximity to the target, which is an important parameter for TRT with ^161^Tb [25]. Exploring the additional radiobiological impact of ^161^Tb on tumor cell membrane would be interesting [26].

To envision the use of Auger-electron emitters for enhanced TRT, the “shuttle” pb12 should be internalized. In living cells, the high affinity [^nat^Tb]Tb-pb12 displayed similar internalization to the endogenous peptide NPY. When radiolabeled with ^111^In, pb12 displayed a time dependent, specific internalization reaching 30% of total binding corresponding to the maximum of hY_1_R internalization capacity, reflecting the full agonist behavior of [^111^In]In-pb12 [27]. Moreover, hY_1_R internalization is rapid and leads to membrane recycling of the receptor [28] illustrated by a significant rapid increase of [^111^In]In-pb12 bound to the cell membrane which stayed constant over the course of 4 h. (Fig. 5).

After hY_1_R-mediated internalization followed by endocytosis [29], our compound was designed to be cleaved by cathepsin B to release the ^111^In-DOTA-NLS unit, which is next addressed to the nucleus after binding to importins. We demonstrated that the recombinant cathepsin-B enzyme effectively cleaved the pb12 sequence (Fig. 2) and this cleavage is necessary to maximize the addressing of ^111^In to the nucleus. In general, cytosol-located molecules with a molecular weight < 45 kDa are able to enter the nucleus via passive diffusion, while bigger molecules require a NLS to be transported by the nuclear pore complex [30]. Therefore, conjugation with NLS moieties was mostly performed for targeted antibodies such as trastuzumab [31]. The NLS conjugation to smaller molecules, as in this study, was also reported to be successful. A trifunctional [^111^In]In-DOTA-NLS-TOC conjugate facilitated nuclear uptake of ^111^In compared with [^111^In]In-DOTA-TOC [18]. In our experiments, we cannot exclude a passive transport of ^111^In (via interactions with membranes as demonstrated above for pb12 and [^nat^Tb]Tb-pb12) into the nucleus, as the addition of the cathepsin B inhibitor did not fully inhibit the nuclear uptake of ^111^In.

Another requirement for TRT radiopeptides is their low efflux rate. Indeed, a high efflux would result in suboptimal irradiation of tumor cells, particularly with short-range particles, and higher off-target dose delivery. [^111^In]In-pb12 demonstrated high efflux which was not inhibited by the efflux inhibitor probenecid. However, using PWR, we demonstrate that compounds pb12 and [^nat^Tb]Tb-pb12 interact with membrane lipids in a non-specific manner (Supplemental Figure 5E and 5F). Taken together, the efflux of [^111^In]In-pb12 we measured probably reflects the association of the peptide with the cell membrane rather than a high efflux, representing a significant improvement compared with truncated peptides [19].

To envision clinical application of our concept, the [^111^In]In-pb12 conjugate needs to remain stable in vivo although full length peptides might be highly sensitive proteases. Despite its palmitic acid modification, known to increase resistance against proteolysis [32], [^111^In]In-pb12 is rapidly metabolized potentially limiting its availability for tumor uptake. In a previous study, [F^7^,P^34^]-NPY conjugated to methotrexate by the GFLG-linker exhibited much higher plasma stability [13]. Therefore, the reason for the fast degradation of [^111^In]In-pb12 is most likely high susceptibility of the arginine/lysine-rich c-Myc NLS (PAAKRVKLD) to proteolysis and stabilization of this sequence is required for further application. We would like to point out that the metabolic stability regarding nucleus targeting studies was never reported in the literature, and this data, although disappointing, is of high interest for laboratories working in this research field. To give more insight in plasmatic stability, we intravenously injected [^111^In]In-pb12 in mice bearing a MCF-7 xenograft. At an early time point, before major radiopeptide degradation, we noted a specific uptake in MCF-7 tumor, confirming the interest of [^111^In]In-pb12 to target the hY_1_R. High accumulation was noted in the liver, consistent with the lipophilicity of the radiopeptide. Another limitation of this work was the low availability of ^161^Tb. Therefore, we performed preliminary experiments with ^nat^Tb which may be only partially representative of the behavior of the ^161^Tb-counterpart as the radiolabeled fraction might be different. In the future, we plan to improve stability of the conjugate and to perform cellular studies comparing the radiotoxicity of different products, including ^111^In, ^90^Y, ^177^Lu, and ^161^Tb-labeled molecules (we are seeking European collaboration to receive ^161^Tb).

## Conclusion

In summary, we have developed a first multifunctional NPY analog allowing nuclear delivery of radiolanthanides. Our results suggest that the sequential mode of action of the construct works, i.e., hY_1_R binding with high affinity and selectivity, hY_1_R-mediated internalization, low efflux, cathepsin B-mediated cleavage of the multifunctional NPY analog, and finally nuclear delivery of the radiolanthanide. However, increasing metabolic stabilization is now needed for future application.

## Supplementary information

**Additional file 1.** Analytical data, supplemental methods, characterization of MCF-7 cells and affinity values of pb13, [^nat^Tb]Tb-pb13, [^111^In]In-pb12, [^111^In]In-pb13, pb12 and [^nat^Tb]Tb-pb12 are presented in Additional file 1.

## Data Availability

The datasets used and/or analyzed during the current study are available from the corresponding author on reasonable request
