# Supplementary material for: Design, synthesis, and biological evaluation of a multifunctional neuropeptide-Y conjugate for selective nuclear delivery of radiolanthanides
Source: EJNMMI Res. 2020 Mar 2;10:16. doi: 10.1186/s13550-020-0612-8 (PMC7052099; doi:10.1186/s13550-020-0612-8)
Supplement: Supplementary file 1 — Additional file 1. Analytical data, supplemental methods, characterization of MCF-7 cells and affinity values of pb13, [natTb]Tb-pb13, [111In]In-pb12, [111In]In-pb13, pb12 and [natTb]Tb-pb12 are presented in Additional file 1. [file 13550_2020_612_MOESM1_ESM.docx]

Supporting informations

EXPERIMENTAL SECTION

CHEMISTRY

Materials for peptide synthesis

9-Fluorenylmethoxycarbonyl (Fmoc)- and *tert*-butyloxycarbonyl (Boc)-protected amino acids were purchased from Orpegen OPC (Heidelberg, Germany), Iris Biotech (Marktredwitz, Germany) and Sigma-Aldrich (Taufkirchen, Germany). NovaSyn TGR R resin was obtained from Novabiochem (Darmstadt, Germany), DOTA-tris(*t*Bu)ester was from CheMatech (Dijon, France) and 1-hydroxybenzotriazole (HOBt), *N,N'*-diisopropylcarbodiimide (DIC) and ethyl 2-cyano-2-(hydroxyimino)acetate (Oxyma) were purchased from Iris Biotech. Dimethylformamide (DMF) and dichloromethane (DCM) were obtained from Biosolve (Valkenswaard, The Netherlands) and acetonitrile (ACN) was from VWR (Darmstadt, Germany). Palmitic acid, dimethylsulfoxide (DMSO), hydrazine, piperidine, thioanisole (TA), trifluoroacetic acid (TFA), terbium(III) chloride hexahydrate and 1-octanol were purchased from Sigma-Aldrich. Thiocresole (TC) was from Alfa Aesar (Ward Hill, MA, USA), diethyl ether was obtained from Merck (Darmstadt, Germany) and ammonium acetate was purchased from Fluka (Buchs, Switzerland).

Synthesis of NPY conjugates

Synthesis of the NPY conjugates was accomplished by a combination of automated solid phase peptide synthesis (SPPS) using a SYRO I peptide synthesizer (MultiSynTech, Bochum, Germany) and manual SPPS. The Fmoc/tert-butyl (Fmoc/tBu) strategy was applied and NovaSyn TGR R resin (15-µmol scale, 0.19 mmol/g) was used to obtain peptides with C-terminal amidation. Amino acids were N-α-Fmoc-protected, except of the N-terminal amino acid of the peptide conjugates, which was N-α-Boc-protected. Full-length peptide backbones Boc-[K^4^(Dde),F^7^,P^34^]-NPY and Boc-[K^4^(Dde),F^7^,A^33^,P^34^,A^35^]-NPY were prepared by automated SPPS. In the automated synthesis, an 8-fold molar excess of amino acid was coupled with equimolar amounts of Oxyma and DIC (8 equiv, 120 µmol) in DMF for 2 × 40 min. The N-terminal Fmoc protecting group was cleaved with 40 % (v/v) piperidine in DMF for 3 min and again 20 % (v/v) piperidine in DMF for 10 min. Removal of the 4,4-dimethyl-2,6-dioxocyclohex-1-ylidenethyl (Dde) protecting group at either the α-amino or ε-amino group of lysine residues was performed with 3 % (v/v) hydrazine in DMF for 10 × 10 min. Manual coupling of amino acids (5 equiv, 75 µmol) was performed with equimolar amounts of HOBt and DIC (5 equiv each) in DMF for at least 2 h. Manual Fmoc deprotection was accomplished with 20 % piperidine in DMF for 2 × 10 min. DOTA-tris(tBu)ester (3 equiv, 45 µmol) was coupled manually with HOBt and DIC (5 equiv each) in DMF overnight. Palmitic acid (5 equiv, 75 µmol) was coupled manually with HOBt and DIC (5 equiv each) in DMF for at least 4 h.

Cleavage of the peptides from the resin and simultaneous side chain deprotection was accomplished using a mixture of TFA/TA/TC (90:5:5, *v*/*v*) for 3.5 h. The crude peptides were precipitated from ice-cold diethyl ether, dissolved in ACN/H_2_O, filtered through a 0.22-µm PVDF filter and subsequently lyophilized. Purification of the crude peptides was accomplished by preparative reversed phase (RP)-HPLC using a Phenomenex Aeris® 5u XB-C18 (250 mm × 21.2 mm, 5 μm, 100 Å) column with a flow rate of 15 mL/min, linear gradients of eluent B (0.08 % (*v*/*v*) TFA in ACN) in eluent A (0.1 % (*v*/*v*) TFA in water) and detection at λ = 220 nm. The purity of the peptides was determined by analytical RP-HPLC using a Phenomenex Jupiter® 4u Proteo C12 90 Å (250 mm × 4.6 mm, 4 μm, 90 Å, 0.6 mL/min) and a Phenomenex Aeris® Peptide 3.6u XB-C18 (250 mm × 4.6 mm, 3.6 µm, 100 Å, 1.55 mL/min) or Agilent VariTide RPC (250 mm × 4.6 mm, 6 µm, 200 Å, 1.0 mL/min) column. A linear gradient of 20%-70% eluent B in eluent A over 40 min was applied and chromatograms were recorded at λ = 220 nm. For all peptides, a purity of > 95 % was obtained. MALDI-ToF mass spectrometry (UltraflexIII, Bruker, Bremen, Germany) and ESI Orbitrap (Orbitrap Elite, Thermo Scientific) mass spectrometry were used to confirm the correct identity of the purified peptides.

Materials for biological methods

Dulbecco's modified Eagle's medium (DMEM), Dulbecco's phosphate buffered saline (DPBS), Ham's F12, trypsin/EDTA and Hank's balanced salt solution (HBSS) were purchased from Lonza (Basel, Switzerland). Penicillin-streptomycin and RPMI 1640 were obtained from Gibco/Life Technologies (Carlsbad, CA, USA). Hygromycin B was purchased from InvivoGen (San Diego, CA, USA), G418-sulfate was from Merck and fetal calf serum (FCS) was obtained from Biochrom (Berlin, Germany) or Gibco/Life Technologies. Opti-MEM was purchased from Life Technologies, Hoechst 33342 and LiCl were purchased from Sigma-Aldrich and BIBP3226 was obtained from Tocris Bioscience (Bristol, UK).

Cell culture

All cell lines were maintained under humidified atmosphere at 37 °C and 5 % CO2 in 75 cm2 cell culture flasks. HEK293 cells stably expressing the hY_1_R or hY_2_R, both C-terminally fused to the enhanced yellow fluorescent protein (eYFP), were cultured in DMEM/Ham's F12 (1:1, v/v) supplemented with 15 % (v/v) FCS and hygromycin (100 µg/mL). COS-7 cells, stably co-transfected with either the hY1R or hY2R, C-terminally fused to eYFP, and the chimeric G protein GαΔ6qi4myr, were cultured in DMEM high glucose supplemented with 10 % (v/v) FCS, hygromycin B (133 µg/mL) and G418-sulfate (1.5 mg/mL). MCF-7 cells and HEK 293 cells (not expressing the hY_1_Rwere cultured in RPMI 1640 supplemented with 10 % (v/v) FCS and penicillin-streptomycin (100 µL/mL).

Western Blot

Protein samples (80µg) were denatured for 5 minutes at 90°C and then loaded onto 10% SDS polyacrylamide gels. Following electrophoresis, proteins were transferred onto nitrocellulose membranes (Immobilon®-P ). Membrane was blocked with 5% non-fat milk at room temperature for 1h, and incubated for 1h at room temperature with the following primary antibodies : Anti-Y_1_ (1:100), Anti-Cathepsine B (1:800) and anti-actine (A2066; Sigma-Aldrich®) (1:5000). Membranes were subsequently incubated with anti-Rabbit IgG (111-035-144 ; Jackson Immunoresearch®) peroxidase-conjugated secondary antibodies (1:10.000) or anti-goat IgG (D1118 ; Santa Cruz Biotechnology®) peroxidase-conjugated secondary antibodies (1:10.000), at room temperature for 45min. Finally, the immunoreactive bands were visualized using ECL Western Blotting detection reagents (RPN2209 ; GE Healthcare®). Semi-quantitative analysis was conducted using ImageJ © (V1.52c) software package to measure densitometric values for each band.

Immunofluorescence

Cells were seeded onto glass cover slips and incubated for 24 hours with complete culture medium. The cells were then rinsed with 2mL of DPBS (14190-094 ;Gibco®), and fixed with 3% paraformaldehyde for 90 minutes. Before the experiment, cells were rinsed three times during 10 minutes with DPBS. The slides were saturated for 90 minutes with PBS/Triton (127K0048 ; Sigma-Aldrich®) 0,3% / BSA (A2153 ; Sigma-Aldrich®) 1% (50µL) and then set in the damp chamber overnight with the primary antibody (Anti-Y_1_; GTX54639 ; GeneTex®) or (Anti-Cathepsin B; FWN0217081 ; R&Dsystems®) (1/100) and PBS/Triton 0,3% / BSA 1%. Cells were then rinsed three times during 10 minutes with DPBS and then placed in the damp chamber, protected from light, with the secondary antibody (1/500) and PBS/Triton 0.3%/BSA1% (50µL). The glass cover slips were rinsed once more with DPBS for three times 10 minutes and then mounted on microscope sliders with Prolonlog (P36931; Invitrogen®) and DAPI (1/5000). All images obtained were analyzed using ImageJ© (V1.52c) software package.

Cathepsin B Human Elisa Assay

A standard curve (from 156 to 10.000ng/mL) for multiple measurements was prepared in triplicate. MCF7 samples were then studied in triplicate (1/2; 1/10; 1/100), according manufacturer instruction to verify the presence and activity of cathepsin-B.

Cell fragment immobilization in the PWR sensor and ligand titration

The protocol for adhesion of cell fragments on the PWR sensor (silica outlayer) was adapted from reported work of Vogel and collaborators,^39^ and has been thoroughly detailed herein.^35^ Briefly, the sensor silica surface was washed with ethanol, and cleaned and activated by plasma cleaner (Diener electronic, Ebhausen, Germany) for 2 min. The sensor silica surface was incubated with a polylysine solution (0.1 mg/mL) for 40 min, followed by a wash with PBS buffer. MCF-7 cells grown to less than 50 % confluence are washed with PBS and covered with water to induce osmotic swelling of the cells. Subsequently, the glass coverslip of the sensor is placed directly onto the cells. Pressure is applied for about 1 min on the glass slide or prism to induce cell rupture and capture of cell fragments. Afterwards, they are removed, ripping off cell fragments containing especially the upper membrane. The sensor is washed with PBS to remove cell debris and maintained in buffer to prevent drying and loss of membrane protein activity. The PWR cell sample (a teflon block with a volume capacity of 250 μL) is placed in contact with the sensor containing the immobilized cell fragments and filled with PBS. After cell fragment deposition, there are positive shifts in the resonance minimum position that are correlated with the total occurring mass gain. We have observed spectral shifts that correlate with those observed for the deposition of lipid model membrane. In the case presented here, some areas of the sensor are covered with cell membranes and others are uncovered. The PWR signal takes into account both covered and uncovered areas as the laser spot is about 0.5 mm in diameter. At the same time, covered areas include both lipids and proteins, meaning that they possess a higher mass than that of a pure lipid membrane. Following stabilization of the signal (no changes in the resonance minimum position with time), peptide ligand is added in an incremental fashion to the chamber and spectral shifts are followed with time. Ligand affinity to the hY_1_R in the MCF-7 cell membrane fragments is calculated by plotting the shifts in the resonance minimum position as a function of ligand concentration and performing an one-site saturation binding fir (Graph Pad Prism). Since between independent experiments the mass of cell membrane fragments and hence receptor quantities vary, which is reflected in the magnitude of the ligand-induced spectral changes, the data is normalized relative to the spectral shifts observed due to cell fragment deposition. Control experiments to investigate ligand binding to cell membrane components other than the receptor (e.g. lipids, sugars) are performed on cell membrane fragments from HEK293 cells that do not express the hY_1_R.

Determination of hY_1_R receptor saturation binding by plasmon waveguide resonance (PWR) spectroscopy

PWR was used to follow receptor conformational changes upon ligand addition to cell membrane fragments overexpressing the hY_1_R that were immobilized in the sensor surface as described below [6]. PWR measurements were performed in a homemade instrument, functioning at a fixed wavelength of 632 nm and variable incident angle with an angular resolution of about 0.5 millidegrees [7]. The polarization angle of the incident light is placed at 45° to allow both p- (parallel to the incident light and perpendicular to the sensor surface) and s-polarized (perpendicular to the incident light and parallel to the sensor surface) light resonances to be obtained within a single angular scan. The sensor consists of a BK-7 prism that is coated with silver and silica to support waveguide modes [7]. All measurements were performed at 22 °C. After cell membrane fragment immobilization, ligand binding was measured by titrating with incremental ligand addition and measuring resonance shifts in both polarizations with time. The system was let to equilibrate before each incremental ligand addition. PWR being sensitive to the optical properties of material deposited on the resonator surface (*i.e.*, of ligand bound to the membrane fragments in the sensor), interference from the material present in the bulk solution (non-bound) is unlikely. Apparent dissociation constants (Kd) were obtained by plotting the resonance minimum position as a function of the peptide concentration and by fitting the plot through a hyperbolic binding function using GraphPad Prism™ version 5.0a (GraphPad Software, San Diego, California, US).

Inositol monophosphate accumulation (IP-One) assay

Receptor activation by non-radioactive peptides was tested in an IP-One assay. COS-7 cells, stably co-transfected with hY_1_R-eYFP or hY_2_R-eYFP and a chimeric G-protein (Gα_∆6qi4myr_), were seeded in a white 384-well plate (6000-7500 cells/well) and grown for 24 h under humidified atmosphere at 37 C and 5 % CO_2_. On the following day, the medium was removed by flipping the plate upside-down and the cells were stimulated with increasing concentrations of peptide in HBSS supplemented with 20 mM LiCl for 1 h at 37 °C and 5 % CO_2_. Peptides were tested in a concentration range from 10^-5^ M to 10^-13^ M in triplicates. Generated inositol monophosphate was subsequently quantified by using the Cisbio IP-One Gq HTRF assay kit according to the manufacturer's instructions. For the assay read-out, a Tecan Spark microplate reader was used. Data analysis was performed with GraphPad Prism 5.03. Obtained HTRF values for the compounds were normalized to NPY and EC_50_ and pEC_50_ values were calculated from sigmoidal concentration-response curves. Each peptide was tested at least two times independently.

Receptor internalization studies

HEK293 cells stably transfected with the hY_1_R or hY_2_R, *C*-terminally fused to eYFP, were seeded into ibiTreat 8-well µ-slides (ibidi, Martinsried, Germany) at a density of 300,000 cells/well and cultured overnight until the cells reached confluency. The culture medium was aspirated and the cells were starved in 200 µL Opti-MEM® with 1 µL nuclear stain Hoechst 33342 (0.5 mg/mL) for 30 min under humidified atmosphere at 37 °C and 5 % CO_2_. For the non-stimulated cell control, the starving solution was aspirated and 200 µL Opti-MEM® were added. For testing the receptor internalization of non-radioactive NPY conjugates, the starving medium was aspirated and 200 µL Opti-MEM® containing 100 nM peptide (hY_1_R) or 1 µM peptide (hY_2_R) were added to the cells. After 1 h of stimulation at 37 °C, the cells were washed and maintained in Opti-MEM®. For fluorescence image acquisition, a Zeiss Axio Observer microscope with an ApoTome.2 Imaging System and a 63× immersion oil objective was used. The nuclear stain Hoechst 33342 was visualized by the DAPI filter (excitation 335−383 nm; emission 420−470 nm) and the eYFP-tag on the receptor by the YFP filter (excitation 488−512 nm; emission 520−550 nm). Image processing was performed with Zeiss ZEN 2 software.

Lipophilicity

The lipophilicity of radiolabeled peptides was assessed by the water-octanol partition/distribution coefficient method. In a centrifuge tube, 500 µL of 1-octanol was added to 500 µL of phosphate-buffered saline (pH 7.4 or pH 5) or water containing the radiolabeled peptide (50 kBq). After equilibrium, the solution was vigorously stirred for 5 min at room temperature and subsequently centrifuged (4000 rpm, 5 min) to yield two immiscible layers. Aliquots of 200 µL were taken from each layer and the radioactivity in the samples was determined by a gamma counter (Perkin Elmer, Waltham, MA, USA).

Metabolic stability

2 MBq of ^111^In-labeled NPY conjugate **3c** were incubated in 2 mL of human blood plasma at 37 °C. At designated time points, samples of 50 µL were taken and 100 µL ethanol was added to precipitate the plasma proteins. The mixture was centrifuged (5 min, 13000 rpm), 50 µL of supernatant were diluted with 150 µL water and analyzed by UV-radio RP-HPLC. The quantity of intact peptide was referred to the control at 0 h (set at 100%). The experiment was performed two times independently.

Membrane integrity assay

For cytotoxicity assay, MCF-7 cells were seeded at a density of 10 000 cells per well (5µL) in 96-well plates and incubated overnight with 95µL of medium (RPMI1640 containing 10% FBS, penicillin-streptomycin (100μg/mL)). Approximately 10^-8^M of the respective pb12, ^nat^Tb-pb12 and control peptide pb13 were added to the medium, and the cells were incubated in triplicates for 4.5 h at 37 °C. Plates were then removed from the incubator and equilibrated to room temperature (approximately 20 minutes). Then, 100µL of Cytotox-One reagent was added to each well, shaken for 30 seconds and incubated for 10 minutes at room temperature. Stop solution (50µL) was added to each well and shaken for 10 seconds. Fluorescence was measured in a plate reader with an excitation wavelength of 560nm and an emission wavelength of 590nm.

Particle Induced X-ray Emission (PIXE) of [^nat^Tb]Tb-pb12

Cell monolayers were prepared and freeze-dried for ion beam analysis according to published protocol [8]. Nuclear microprobe analysis of intracellular chemical element contents was performed at the AIFIRA facility, using a 3.0 MeV proton beam focused onto the sample surface, ending with a beam diameter of 1 µm and a beam current of approximately 100 pA [9]. The beam was continuously scanned over square-shaped regions of interest of approximately 150 µm in width. Typical acquisition time for a single analysis was 1 to 2 hours. X-rays emitted after sample-beam interaction were measured using 2 Si(Li) x-ray photon detectors symmetrically placed at 20 mm from sample surface with an angle of 45°. X-ray detectors were covered with a carbon “funny” filter (filter with a pinhole) in order to prevent backscattered protons to blind them. The number of protons delivered during an acquisition, as used for quantification, was measured at the same time by RBS analysis using a PIPS (passivated implanted planar silicon) detector (Canberra PD 50-11-300 AM) placed at a 135° from incoming beam direction. A set of reference samples (GdF_3_, CuS, Fe and ZnTe) with certified elemental concentration (100 µg/cm² ± 5%) was used to measure quantification accuracy of the overall set-up (beam geometry – detectors configuration – filters). Calibration of our experimental set-up resulted in concentration measurements in agreement with certified values for element above potassium (X-ray energy > 3.7 keV). Lighter elements producing X-ray photons with lower energy cannot be quantified with a reasonable accuracy due to photons absorption by the funny filter. Results are expressed in percentage of limit of detection.

**RESULTS**

PEPTIDE SYNTHESIS

Analytical data of all compounds studied in this work are summarized in Supplemental table 1. Exemplary analytical data is shown for conjugate pb12 and [^nat^Tb]Tb-pb12 in Supplemental figure 1 below.


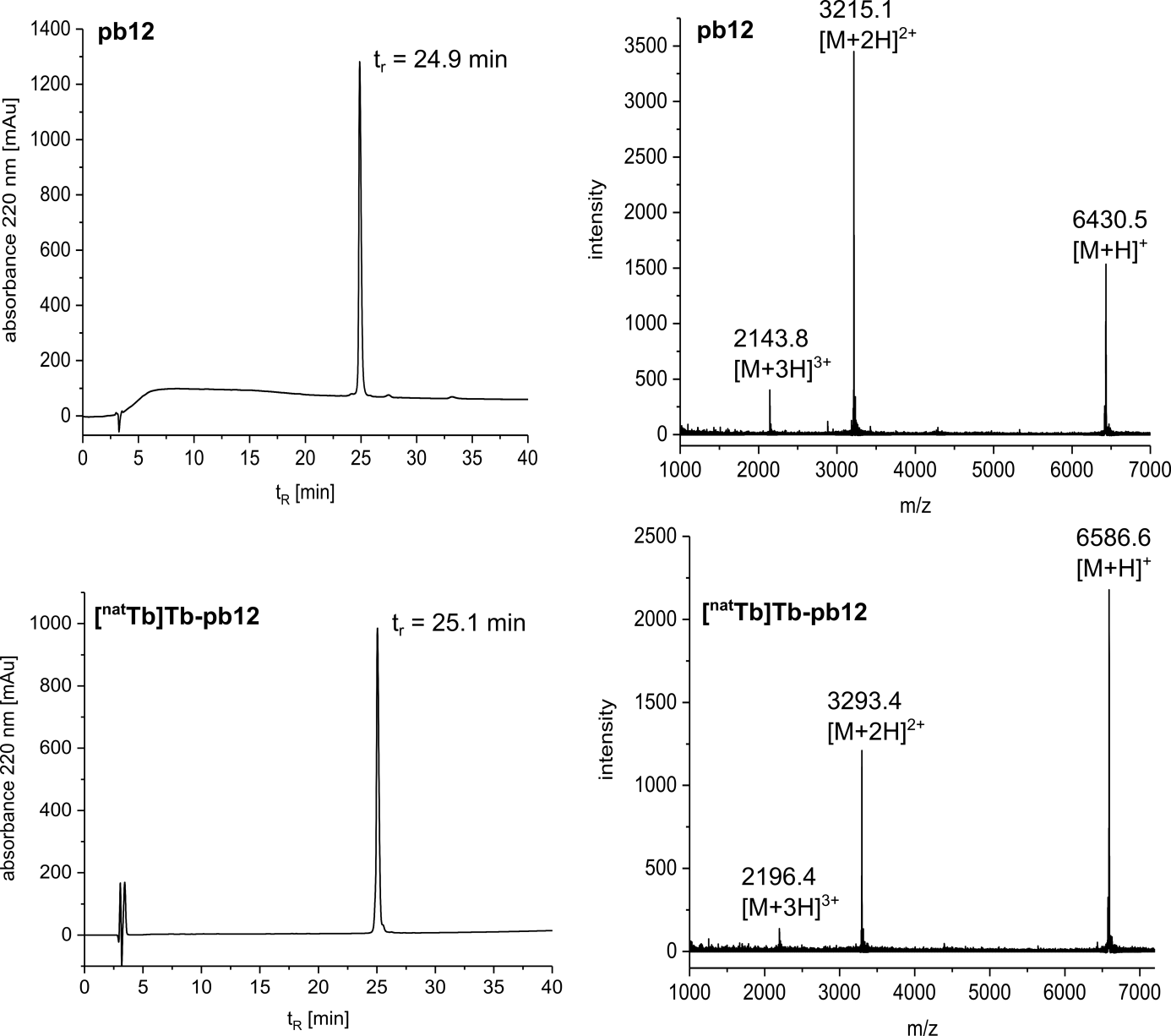


Supplemental figure 1: Analytical data of the peptide conjugate pb12 and the ^nat^Tb-labeled version [^nat^Tb]Tb-pb12. Top left: RP-HPLC analysis of pb12 using an Agilent VariTide RPC 200 Å column and a linear gradient of 20%-70% (*v/v*) eluent B in eluent A over 40 min. Eluent A: H_2_O + 0.1 % (*v/v*) TFA, eluent B: acetonitrile + 0.08 % (*v/v*) TFA. Top right: MALDI-ToF mass spectrum showing the single-, double- and triple-charged molecular ion species of pb12. M_exact_ (calc.): 6429.5 Da. Bottom left: RP-HPLC analysis of ^nat^Tb-labeled [^nat^Tb]Tb-pb12 using an Agilent VariTide RPC 200 Å column and a linear gradient of 20%-70% (*v/v*) eluent B in eluent A over 40 min. Bottom right: MALDI-ToF mass spectrum showing the single-, double- and triple-charged molecular ion species of ^nat^Tb-labeled pb12. M_exact_ (calc.): 6585.4 Da.

| compound | Analyt. RP-HPLC | | MALDI-ToF MS | | purity |
| --- | --- | --- | --- | --- | --- |
|  | t_R_^1^ [min]^a^ | t_R_^2^ [min] | M_exact_ (calc.)^d^  [Da] | M_exact_ (exp.)^e^  [M+H]^+^ |  |
| NPY | 21.3 | 16.2^b^ | 4251.1 | 4252.1 | > 95 % |
| [F^7^,P^34^]-NPY | 21.0 | 15.2^b^ | 4253.1 | 4254.1 | > 95 % |
| pb12 | 27.1 | 24.9^c^ | 6429.5 | 6430.5 | > 95 % |
| [^nat^Tb]Tb-pb12 | - | 25.1^c^ | 6585.4 | 6586.6 | > 95 % |
| pb13 | 28.5 | 25.4^c^ | 6259.4 | 6260.3 | > 95 % |
| [^nat^Tb]Tb-pb13 | - | 25.1^c^ | 6415.3 | 6416.4 | > 95 % |

Supplemental Table 1. Analysis of synthesized peptides by analytical RP-HPLC and MALDI-ToF MS.

^a^Retention time t_R_^1^ was determined on a Phenomenex Jupiter® 4u Proteo C12 90 Å column using a linear gradient of 20%-70% eluent B in eluent A over 40 min. ^b^Retention time t_R_^2^ for NPY and [F^7^,P^34^]-NPY was determined on a Phenomenex Aeris® Peptide 3.6u XB-C18 column using a linear gradient of 20%-70% eluent B in eluent A over 40 min. ^c^Retention time t_R_^2^ for pb12, [^nat^Tb]Tb-pb12, pb13 and [^nat^Tb]Tb-pb13 was determined on a Agilent VariTide RPC 200 Å column using a linear gradient of 20%-70% eluent B in eluent A over 40 min. ^d^M_exact_ (calc.): calculated monoisotopic mass in Dalton. ^e^M_exact_ (exp.): experimentally measured monoisotopic mass.

CHARACTERIZATION OF MCF-7 CELLS

Western Blot

Immunoblots confirmed that MCF-7 cells effectively express the hY_1_R (designated as Y_1_ on the Supplemental Figure 2 below) and the cathepsin B enzyme. They are suitable cells to investigate our NPY-conjugates.


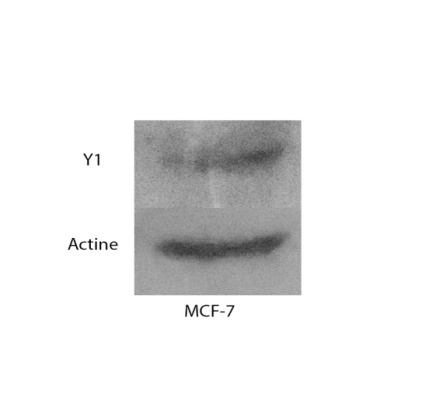

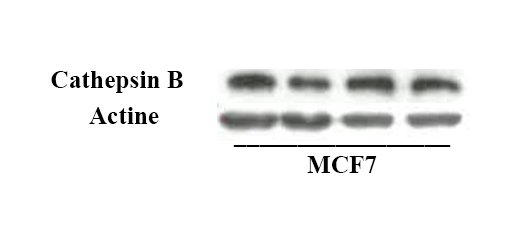


Supplemental Figure 2: Western Blot of hY_1_R and cathepsin-B in MCF-7 cells

Immunofluorescence

Using immunofluorescence experiments, we confirmed that MCF-7 cells expressed the hY_1_R. Moreover, a membranous labelling was seen for hY_1_R as expected with G-protein coupled receptor.


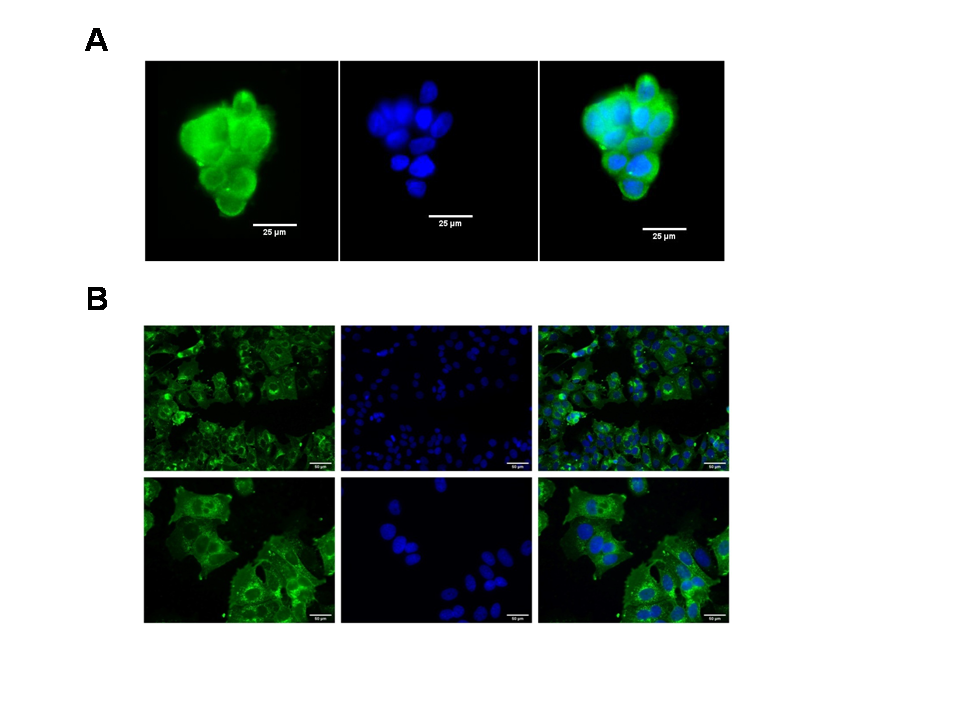


Supplemental figure 3: Immunofluorescence of hY_1_R and cathepsin-B in MCF-7 cells at magnification X20. A – left image: subcellular localization of hY_1_R assessed by immunofluorescence. Membranous hY_1_R expression is seen on MCF-7 cells (green). Middle: DNA staining with DAPI (blue). Right image: fused image. B – left image: subcellular localization of cathepsin-B assessed by immunofluorescence. Granular cytoplasmic staining of cathepsin-B is seen on MCF-7 cells (green). Middle: DNA staining with DAPI (blue). Right image: fused image. Scale bar = 25µm

Cathepsin B Human Elisa Assay

Using ELISA, cathepsin B activity was found to be 57.11 ± 6.13 ng/mL confirming that MCF-7 cells could be used to study our NPY-conjugates metabolized by the cathepsin B.


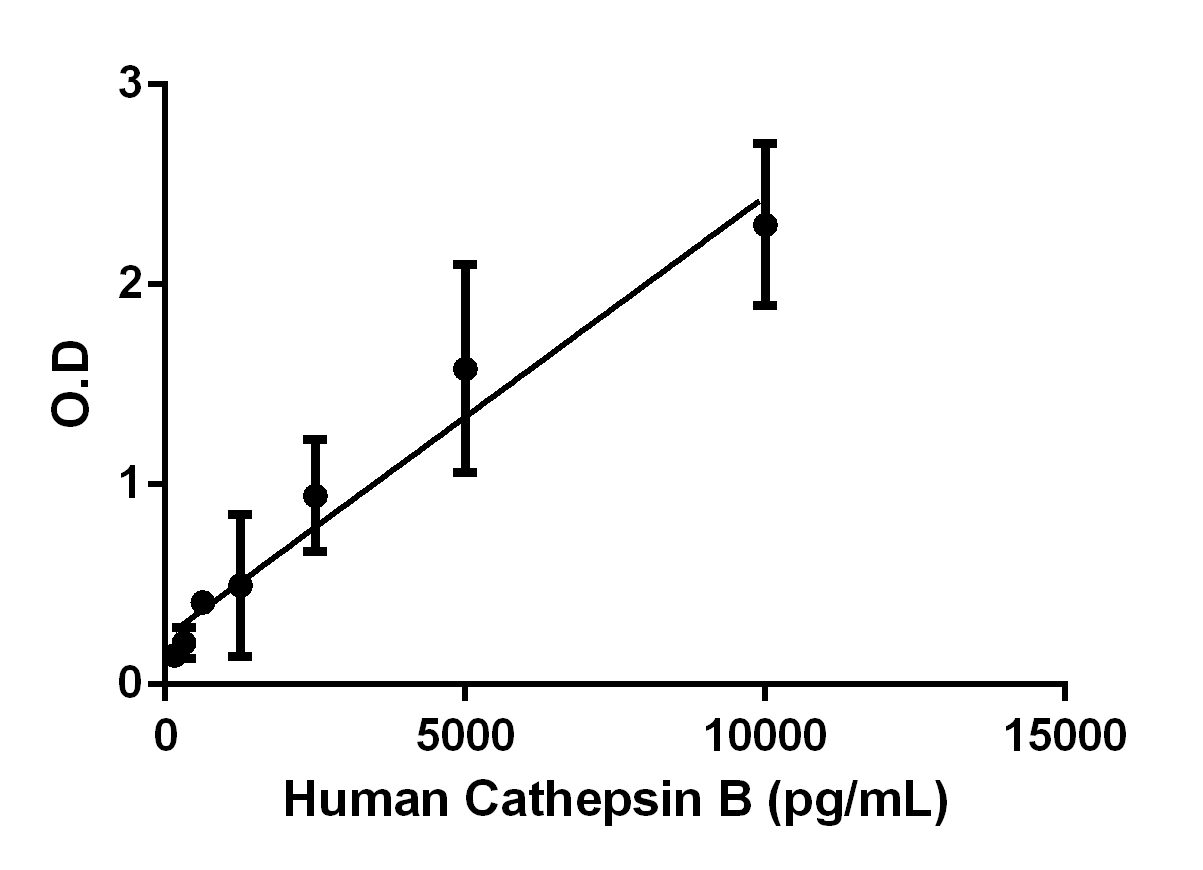


Supplemental Figure 4: ELISA experiment to determine cathepsin B activity in MCF-7 cells. O.D stands for optical density.

All together, these approaches confirm that MCF-7 cells possess all biological equipment (hY_1_R and active cathepsin-B enzyme) suitable to study our NPY-multivalent compound.

Affinity values of pb13, [^nat^Tb]Tb-pb13, [^111^In]In-pb12, [^111^In]In-pb13, pb12 and [^nat^Tb]Tb-pb12


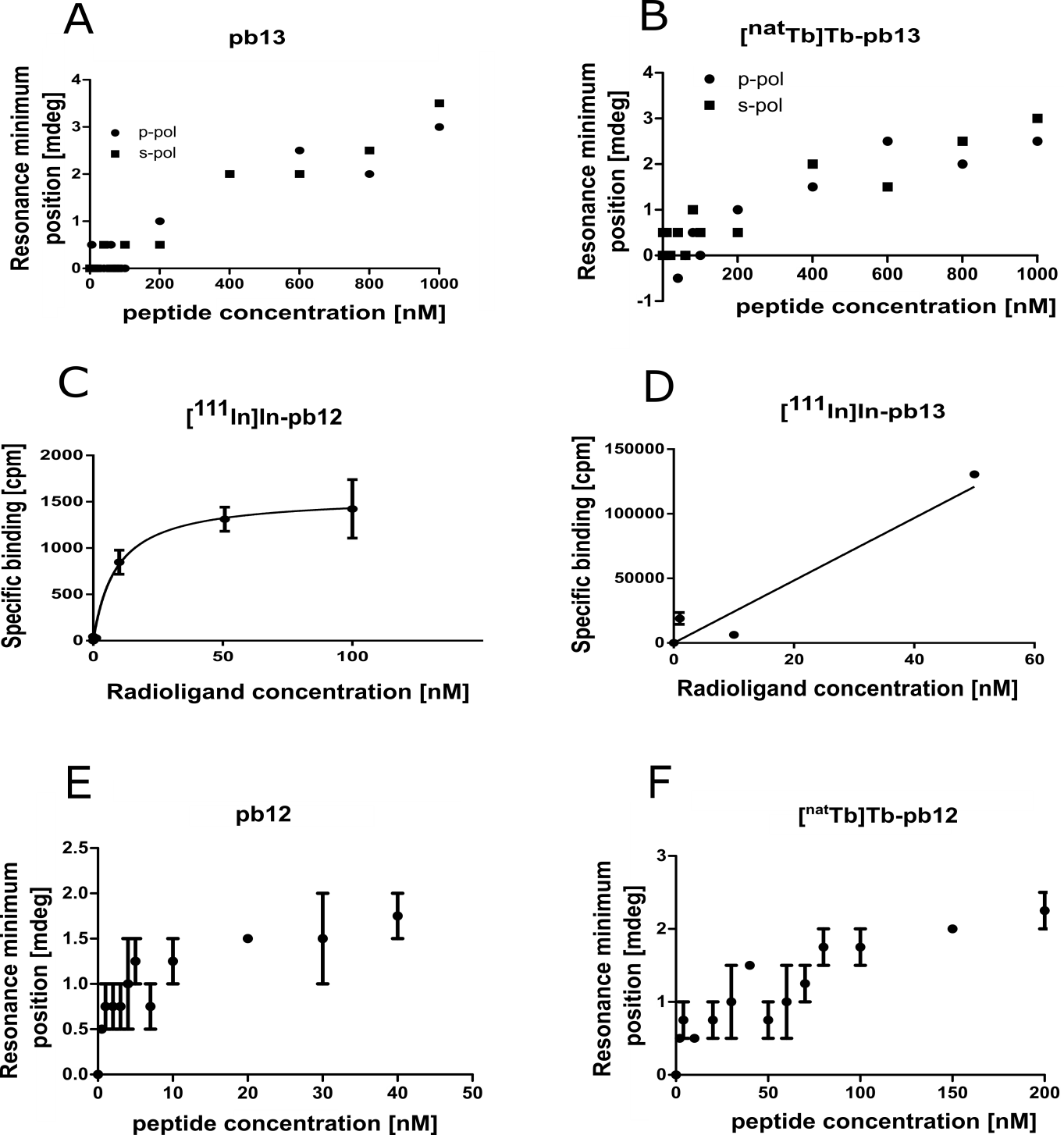


Supplemental figure 5. Affinity values of some NPY-conjugates developed in this work to the hY_1_R determined by PWR (A, B, E, F) and radiolabelling assay (C, D). The unlabeled control peptide pb13 (A), the terbium-labelled control peptide [^nat^Tb]Tb-pb13 (B) and the radiolabeled control peptide [^111^In]In-pb13 (D) showed no specific binding to MCF-7 fragments The [^111^In]In-pb12 (C) exhibit specific binding to MCF-7. As additional control experiments, pb12 (E) and [^nat^Tb]Tb-pb12 present some non-specific interaction to hY_1_R-negative HEK293 cells.

REFERENCES

1. Böhme D, Beck-Sickinger AG. Controlling toxicity of Peptide-drug conjugates by different chemical linker structures. ChemMedChem. 2015;10:804–14.

2. Worm DJ, Hoppenz P, Els-Heindl S, Kellert M, Kuhnert R, Saretz S, et al. Selective Neuropeptide Y Conjugates with Maximized Carborane Loading as Promising Boron Delivery Agents for Boron Neutron Capture Therapy. J Med Chem. 2019

3. Zhong Y-J, Shao L-H, Li Y. Cathepsin B-cleavable doxorubicin prodrugs for targeted cancer therapy (Review). Int J Oncol. 2013;42:373–83.

4. Dang CV, Lee WM. Identification of the human c-myc protein nuclear translocation signal. Mol Cell Biol. 1988;8:4048–54.

5. Hofmann S, Maschauer S, Kuwert T, Beck-Sickinger AG, Prante O. Synthesis and in Vitro and in Vivo Evaluation of an ^18^F-Labeled Neuropeptide Y Analogue for Imaging of Breast Cancer by PET. Mol Pharmaceutics. 2015;12:1121–30.

6. Boyé K, Billottet C, Pujol N, Alves ID, Bikfalvi A. Ligand activation induces different conformational changes in CXCR3 receptor isoforms as evidenced by plasmon waveguide resonance (PWR). Sci Rep. 2017;7.

7. Harté E, Maalouli N, Shalabney A, Texier E, Berthelot K, Lecomte S, et al. Probing the kinetics of lipid membrane formation and the interaction of a nontoxic and a toxic amyloid with plasmon waveguide resonance. Chem Commun. 2014;50:4168–71.

8. Muggiolu G, Simon M, Lampe N, Devès G, Barberet P, Michelet C, et al. In Situ Detection and Single Cell Quantification of Metal Oxide Nanoparticles Using Nuclear Microprobe Analysis. J Vis Exp. 2018;

9. Sorieul S, Alfaurt Ph, Daudin L, Serani L, Moretto Ph. Aifira: An ion beam facility for multidisciplinary research. Nuclear Instruments and Methods in Physics Research Section B: Beam Interactions with Materials and Atoms. 2014;332:68–73.
